# Use of fractional exhaled nitric oxide as a potential predictor of bronchodilator response

**DOI:** 10.1097/MD.0000000000034073

**Published:** 2023-07-14

**Authors:** Bruna Cuoco Provenzano, Thiago Prudente Bartholo, Kennedy Martins Kirk, Mario Fritsch Neves, Marcelo Ribeiro-Alves, Ana Paula Ramos Barreto, Nadja Polisseni Graça, Paulo Roberto Chauvet Coelho, Claudia Henrique da Costa, Rogerio Rufino

**Affiliations:** a Rio de Janeiro State University, Rio de Janeiro, Brazil; b Fundação Oswaldo Cruz, Rio de Janeiro, Brazil.

**Keywords:** asthma, biomarkers, fractional exhaled nitric oxide testing, monitoring

## Abstract

Nitric oxide (NO) is an important product of eosinophilic metabolism, and its increase is associated with bronchial remodeling and airway hyperresponsiveness. Fractional exhaled NO (FENO) in the expired air of patients with suspected or diagnosed asthma has been used as a marker for eosinophilic inflammation. This cohort study included asthmatic patients classified under steps 3, 4, or 5 of the global strategy for asthma management and prevention. In the morning of the same day, all patients underwent blood collection for eosinophil counts, followed by FENO measurement and spirometry. We considered 2 groups based on the bronchodilation (BD) response on spirometry (>10% of FVC or FEV_1_): positive (BD+) and negative (BD−). Differences between the 2 groups were analyzed for demographic features, FENO values, and predictive correlations between FENO and BD. Both groups of patients showed an increase in the eosinophil count (BD+, *P* = .03; BD−, *P* = .04) and FENO values (*P* = .015 for both) with an increase in the asthma severity from step 3 to step 5 of the global strategy for asthma management and prevention. The correlations of FENO and eosinophils as well as FENO values and BD + were 0.127 (95% confidence interval,–0.269 to –0.486) and 0.696 (95% confidence interval, 0.246–0.899; *P* = .007), respectively. Measuring FENO levels may be useful for identifying patients with BD+.

## 1. Introduction

Nitric oxide (NO) is an important free radical produced during the arginine cycle. It functions as a messenger that interferes with several inflammatory cascades. In patients with asthma, NO is an important product of eosinophilic metabolism, and its increase is related to bronchial remodeling and airway hyperresponsiveness.^[1,2]^ Recently, various technological developments have been made to detect fractional exhaled NO (FENO) in the expired air of patients with suspected or diagnosed asthma.^[3]^ FENO has become a diagnostic indicator and a tool for prognostic and therapeutic evaluations.^[4,5]^ Moreover, the advent of immunobiology has brought great advances in the treatment of asthma, with predominantly immunological and pathophysiological approaches.^[6]^ Therefore, using FENO to identify the predominant inflammatory mechanism has become essential for assessing severely ill patients refractory to inhaled and systemic treatments.^[7]^ However, some gaps are yet to be addressed, such as their correlation with bronchial reactivity and flow variability, which are essential for the diagnosis of asthma. Thus, the main objective of this study was to analyze FENO values in a cohort of patients classified under steps 3, 4, and 5 of the global strategy for asthma management and prevention (GINA)^[8]^ and to correlate these values to positive bronchodilation (BD) results on spirometry. In addition, these findings could identify patients who would potentially demonstrate bronchodilator test positivity.

## 2. Methods

This prospective cohort study included patients treated at the Severe Asthma Center of the Rio de Janeiro State University. The inclusion criteria were age > 18 years, taking ≥ 800 µg of budesonide, and classified under steps 3, 4, or 5 of the GINA 2022. Patients underwent blood collection for eosinophil count, spirometry, and FENO measurements in the morning of the same day. The exclusion criteria were history of smoking and coexisting chronic obstructive pulmonary disease. All participants provided free and informed consent. This study was approved by the Committee for the Ethics of Research (CAAE-94348718.8.0000.5259).

All patients underwent blood collection, followed by 2 FENO measurements and spirometry to minimize the influence of spirometry and the BD maneuver on NO measurements. In addition, an epidemiological questionnaire was used to collect data on variables such as age, sex, time since diagnosis, medications used, number of attacks in the previous year, body mass index (BMI), and asthma control assessment using the GINA questionnaire.^[8]^

Spirometry tests were performed using an HD CPL apparatus (nSpire Health Inc., Longmont, CO) following the American Thoracic Society (ATS) criteria.^[9,10]^ Forced vital capacity (FVC)%, forced expiratory volume in 1 second (FEV1)%, and the FEV1/FVC ratio (%) of predicted were determined before and after 20 minutes of using the inhaled bronchodilator (salbutamol spray, at a dose of 400 µg).^[10]^ BD values were calculated according to the ATS/European Respiratory Society for 2022 and an increment above 10% of the predicted FEV1 or FVC value was considered a response to the bronchodilator.^[9,10]^

FENO levels were measured using the NIOX VERO device according to the technique recommended by the ATS and European Respiratory Society.^[2]^ NO levels were measured in a single breath exhaled directly into the analyzer at 50 mL/s for at least 6 seconds. The exhalation was repeated, and the reproducibility was verified. The exhaled NO levels were measured in parts per million (ppm) and obtained in real time.

The estimated statistical power for pairwise mean differences between groups of 2 repeated measures of FENO were 100% for both Gina step 4 to 5 and sex. Other contrasts had a lower statistical power, between 0.05 and 0.53, assuming paired two-tailed *t* tests with significance levels (type I error probability) of 0.05 and sample sizes (in each group) for the calculation of effect sizes. The Mann–Whitney *U* test was used for continuous numerical variables to compare the demographic, clinical, and immunological characteristics and baseline pulmonary function tests between the BD test groups with negative and positive results. The Fisher’s exact test was used to evaluate the frequency independence between categorical nominal variables and disease severity. For intra-rater agreement in the FENO measurements, the average intraclass correlation coefficient for the two-way random-effects (patients and raters) analysis of variance model (Shrout and Fleiss, 1979) was used as an index of intra-rater reliability, consistency, and agreement. *F* tests and one-sided 95% confidence intervals (CIs) were used to assess statistical significance. Moreover, Bland–Altman plots were drawn to illustrate the 95% CIs for bias and limits of agreement. Pearson’s correlation coefficient was used to compare continuous variables. Multiple linear mixed-effects models were used to evaluate the log-transformed repeated FENO measurements, either between the BD test groups or among the levels of bronchial responsiveness and GINA, and patients were considered random effects. In addition, we introduced any clinical/phenotypic features associated with FENO as confounders to eliminate any possible bias introduced by convenience sampling. The fixed systematic component of the models was adjusted for the confounding variables of age, BMI, sex, time since asthma diagnosis (in years), number of exacerbations in the previous year, severity (GINA steps), and current use of topical corticosteroids. The Tukey honest significant difference method was used to correct p-values using the number of comparisons whenever necessary. Estimated mean marginal effects and their 95% CIs are presented graphically, whereas all other variables in the multiple linear mixed models are presented as means or equal proportions using contrasts constructed from these estimated mean marginal effects. Statistical significance was set at *P* < .05. The software R version 4.1.1 (R Core Team, 2021), packages “lme4” and “emmeans,” and their dependencies were used to perform statistical analysis.

## 3. Results

Outpatients from the Severe Asthma Center were invited to participate and 30 of them were recruited; however, one was excluded due to difficulty in performing the FENO measurement technique. Thus, the data of 29 patients were analyzed; 82% were women and 18% were men. Most patients were obese or overweight (65.5%), with a median age of 43 years and an average time since diagnosis of 30 years. Approximately 41.4% of the patients had GINA step 3, 44.8% had GINA step 4, and 13.8% had GINA step 5. The mean dose of inhaled budesonide was 800 µg/d. The mean number of exacerbations in this cohort in the previous year was 4, and the median concentration of exhaled NO was 34 ppm. Table 1 presents the descriptive analysis (Supplemental Digital Content, http://links.lww.com/MD/J341).

**Table 1:** Measurements and data of patients.

Variables	Total	BD− group	BD+ group	*P* value
Sex				
M	5 (17.2%)	3 (23.1%)	2 (12.5%)	.632*
F	24 (82.8%)	10 (76.9%)	14 (87.5%)	
Age (yr)	43 (IQR = 16)	46 (IQR = 14)	40 (IQR = 15.5)	.665
BMI (kg/m^2^)	28.5 (IQR = 18.0)	30 (IQR = 12.0)	26 (IQR = 18)	.037
Time since asthma diagnosis (yr)	30 (IQR = 20)	35 (IQR = 17)	26 (IQR = 21)	.555
Number of exacerbations (last year)	4 (IQR = 3)	3.5 (IQR = 6)	4 (IQR = 3)	.980
Eosinophil count (/mm^3^)	200.5 (IQR = 161.3)	214.8 (IQR = 187.3)	181.8 (IQR = 124.3)	.975
FENO (ppm)	34 (IQR = 31)	20 (IQR = 29)	38.5 (IQR = 30)	.345
FVC pretest (L)	2.86 (IQR = 0.91)	2.86 (IQR = 0.63)	2.88 (IQR = 0.91)	.844
FVC pretest (%)	89 (IQR = 23)	91 (IQR = 13)	88 (IQR = 29.5)	.796
FEV_1_ pretest (L)	1.96 (IQR = 0.99)	2.01 (IQR = 0.72)	1.77 (IQR = 1.01)	.249
FEV_1_ pretest (%)	67 (IQR = 27)	74 (IQR = 17)	64 (IQR = 40.5)	.201
Step according to GINA (2022)				
Step 3	12 (41.4%)	3 (23.1%)	9 (56.2%)	.0005†
Step 4	13 (44.8%)	8 (61.5%)	5 (31.2%)	
Step 5	4 (13.8%)	2 (15.4%)	2 (12.5%)	

BD− group = group with negative bronchodilation test results, BD+ group = group with positive bronchodilation test results, BMI = body mass index, F = female, FENO = fractional exhaled nitric oxide, FEV1 = forced expiratory volume in 1 s, FVC = forced vital capacity, GINA = Global Strategy for Asthma Management and Prevention, IQR = interquartile range, M = male, ppm = parts per million.

* *F* value = 0.582 (analysis of variance).

† *F* value = 9.700 (analysis of variance).

**Figure 1.**
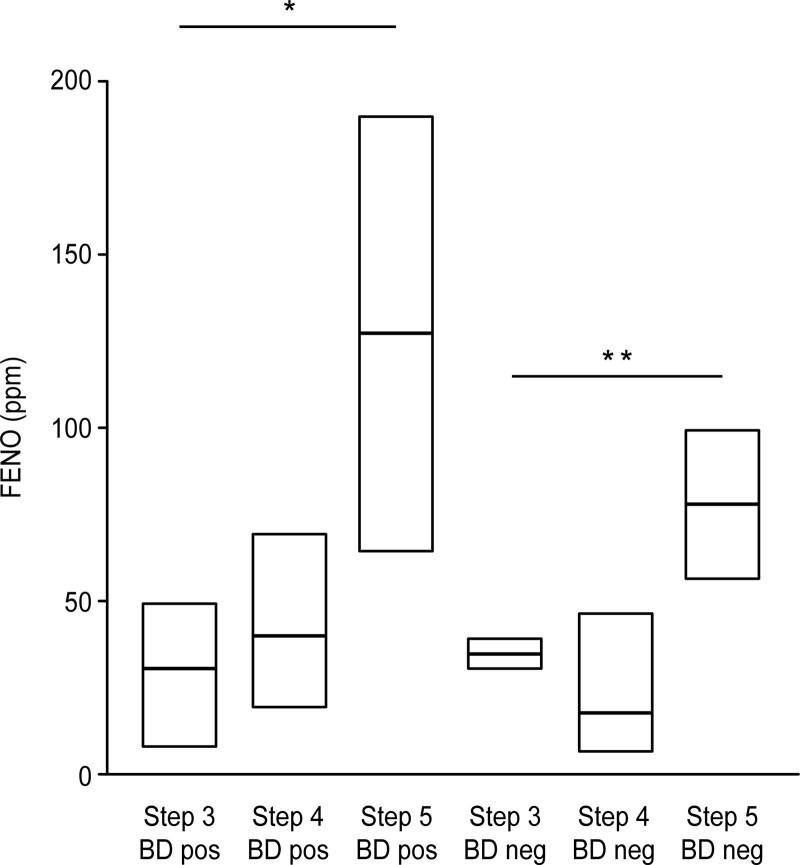
Values of fractional exhaled nitric oxide by severity in patients with asthma. *P = .030 (Mann–Whitney *U* test); **P = .044 (Mann–Whitney *U* test), Steps from Global Strategy for Asthma Management and Prevention (GINA).^[8]^ In both the groups of patients with and without bronchodilation response, FENO values increased with the severity. BD pos = positive bronchodilation test result, BD neg = negative bronchodilation test result, FENO = fractional exhaled nitric oxide, ppm = parts per million.

Statistical analysis of the samples revealed no correlation between the FENO values and the presence or absence of a BD response on spirometry. However, there was a slight tendency for the FENO values to be higher in the BD+ group than in the BD− group (Fig. 1). Furthermore, FENO levels were higher among men than among women (log[male/female] = 1.00, *P* = .03).

**Figure 2.**
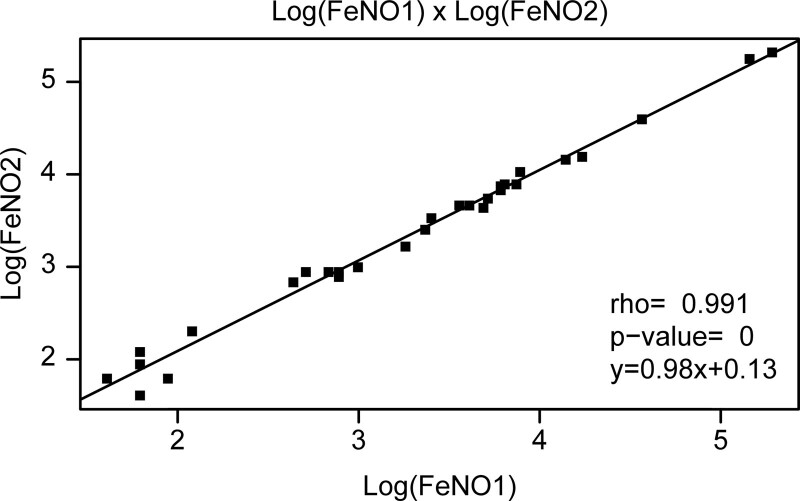
Two sequential fractional exhaled nitric oxide measurements of each patient. There was no difference in the log-transformed FENO values between the first and second measurements. In this sample, almost the same value was obtained in both measurements. FENO1 = first fractional exhaled nitric oxide measurement, FENO2 = second fractional exhaled nitric oxide measurement.

A total of 58 FENO measurements were performed in this cohort of 29 patients. The intraclass correlation coefficient was 99.7% (99.35–99.86; *P* < .001) with a mean agreement of 0.12 (–0.146 to –0.273) between the first and second FENO measurements. Thus, it is acceptable and reasonable to conduct only one measurement for each patient (Fig. 2).

**Figure 3.**
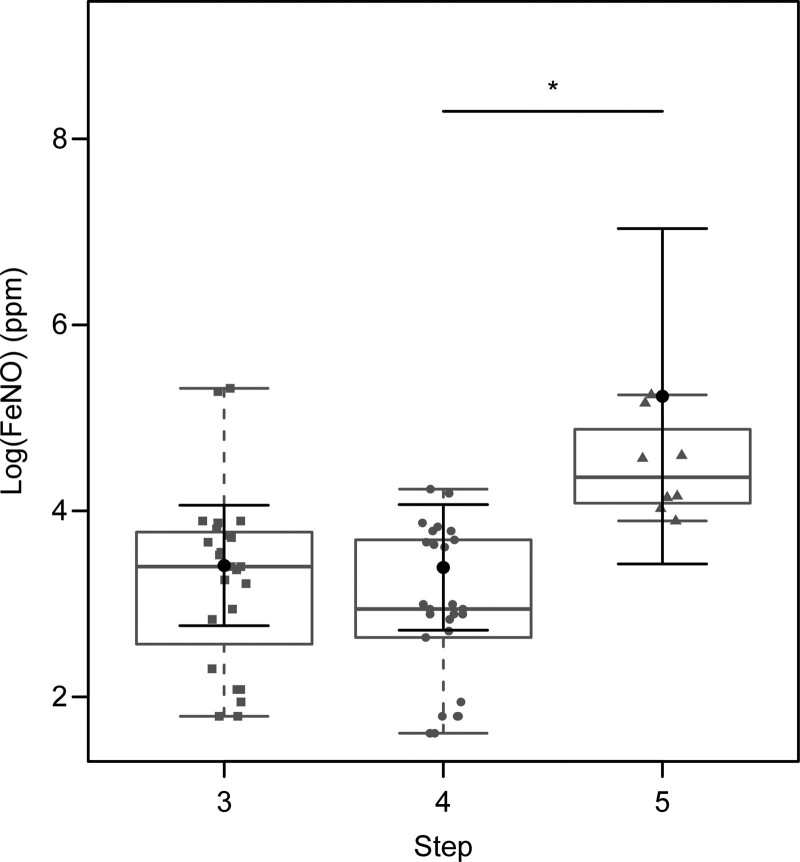
Fractional exhaled nitric oxide values by severity of asthma. Steps from Global Strategy for Asthma Management and Prevention.^[8]^ FENO = fractional exhaled nitric oxide, ppm = parts per million.

After correcting for the confounding variables of age, sex, BMI, time since diagnosis, number of exacerbations in the previous year, severity (GINA Step), and dose of inhaled corticosteroid used, the GINA step 5 patients had higher mean FENO values than the GINA step 4 patients (*P* = .015) (Fig. 3).

**Figure 4.**
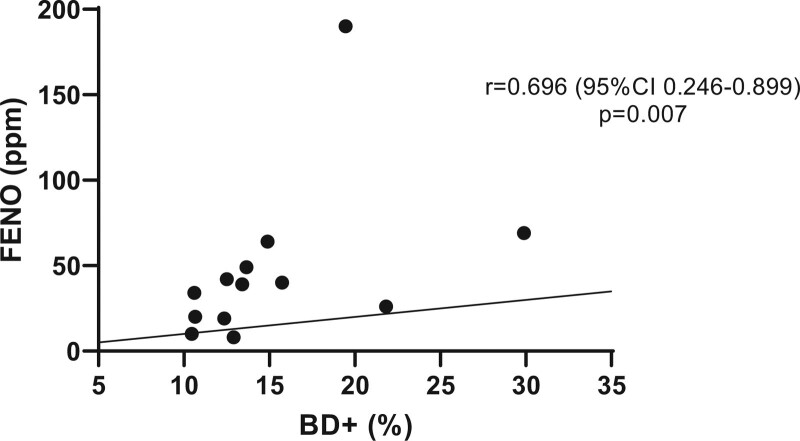
Correlation fractional exhaled nitric oxide and positive response to bronchodilators. The Spearman correlation was performed. BD+ group = group with positive bronchodilation test results, FENO = fractional exhaled nitric oxide.

The cohort was divided into BD+ and BD− groups, comprising patients with positive and negative BD test results, respectively, on spirometry. The BD+ and BD− groups had a mean age of 40 years and 42 years, respectively, and a mean time since diagnosis of 26 and 29 years, respectively. The FENO values were 46.9 and 41.8 ppm, respectively, and predominantly comprised GINA step 3 (56.2%) and step 4 patients (61.5%). There was a strong positive correlation between FENO and BD+ group values (Fig. 4). The correlation of the BD− group with FENO (*P* = .714, *R* = 0.098 [95% CI, −0.431 to 0.5774]) and FENO with all patients (BD+ and BD− values) (*P* = .117, *R* = 0.298 [95% CI, −0.088 to 0.606]) did not demonstrate statistical significance.

## 4. Discussion

Asthma is a chronic inflammatory airway disease with multiple pathophysiological mechanisms.^[8]^ Recently, with the advent of immunobiological drugs, identifying the most prevalent inflammatory pathway has become increasingly important in the improvement of treatment in patients.^[11]^ Thus, biomarkers, such as FENO, help identify the eosinophilic inflammation profile, which is of fundamental importance not only in the diagnostic evaluation but also in the therapeutic follow-up of these patients.^[1,6]^ The findings of this study show that FENO has the potential to help physicians in therapeutic orientation, as high values might be associated with a bronchodilator response.

FENO values must be interpreted while considering certain technical and patient-related aspects.^[5]^ According to the ATS consensus, an FENO level >50 ppm is suggestive of eosinophilic inflammation, with a likely response to inhaled corticosteroids. However, values between 25 and 49 ppm must be interpreted with caution and should be correlated with other biomarkers.^[2,12]^ In this cohort, the mean FENO value was 34 ppm, and higher exhaled NO values were suitable for predicting bronchial reactivity. It is expected that patients with detectable bronchial responsiveness on spirometry would have increased inflammation and high exhaled NO levels.^[13,14]^

The median FENO value in our study was 34 ppm, and according to the ATS,^[12]^ it is not sufficient to establish an eosinophilic inflammation profile. Indeed, this cohort predominantly included patients with a prolonged time of diagnosis who probably had more than one inflammatory mechanism and not necessarily an eosinophilic mechanism. This could have contributed to the lower-than-expected FENO value. Furthermore, the process of bronchoreactivity and BD response is multifactorial and not exclusive to the eosinophilic pathway of inflammation.^[15,16]^ FENO is applicable not only for identifying patients with severe asthma but also for those at a higher risk of exacerbations, serving as a guide for the clinical management of this group of patients.^[3]^

The assessment of FENO in the airways depends on several factors, such as the use of medications and cigarettes.^[1,2]^ In this cohort, FENO values were significantly higher in men than in women despite the small number of participants. The relationship between FENO values and sex is controversial and not well-established. Some studies have shown that women are mainly predisposed to allergic asthma, mostly due to hormonal effects; however, this report contradicts the reports of international literature.^[2,17]^ In contrast, there are cohorts in post hoc analyses that show that NO levels are higher in men than in women.^[1]^

Although the FENO methodology is simple from a practical viewpoint, it requires continuous exhalation at a flow of 0.05 L/min.^[10]^ Despite being well-established for the diagnosis of severe asthma, its use in low-resource developing countries is restricted for financial reasons.^[18]^ This study showed good reproducibility and repeatability of results. Therefore, only one measurement per patient was considered satisfactory. These results are relevant because they demonstrate a reduction in the number of tests conducted and an increase in the availability of tests, regardless of the service expertise in the method.

The main limitation of this prospective cohort study was its small sample size, which affected the distribution of the results and resulted in great variability in the values obtained. Other limitations of the study were that it was carried out in a single center and it used a convenience sample.

Advances in technology and discoveries regarding the pathophysiology of eosinophilic asthma have expanded the scope and applicability of FENO in the evaluation of patients with diagnosed or suspected asthma. Measuring FENO can be helpful in identifying eosinophilic asthma and disease exacerbation as well as predicting the bronchodilator response. However, we observed that FENO was higher in step 4 and 5 patients, even in those who had lower eosinophil values in the peripheral blood. Our findings present the possibility of using FENO to predict BD.

## Acknowledgments

We would like to thank the Pulmonology Service, Department of Chest Diseases, Medical Sciences Faculty, Rio de Janeiro State University, Brazil.

## Author contributions

**Conceptualization:** Thiago Prudente Bartholo, Kennedy Martins Kirk, Mario Fritsch Neves.

**Data curation:** Bruna Cuoco Provenzano, Kennedy Martins Kirk, Ana Paula Ramos Barreto, Nadja Polisseni Graça, Paulo Roberto Chauvet Coelho.

**Formal analysis:** Thiago Prudente Bartholo, Marcelo Ribeiro-Alves, Rogerio Rufino.

**Funding acquisition:** Claudia Henrique da Costa, Rogerio Rufino.

**Investigation:** Bruna Cuoco Provenzano, Kennedy Martins Kirk, Mario Fritsch Neves, Ana Paula Ramos Barreto, Nadja Polisseni Graça, Paulo Roberto Chauvet Coelho.

**Methodology:** Thiago Prudente Bartholo, Kennedy Martins Kirk, Mario Fritsch Neves, Marcelo Ribeiro-Alves, Claudia Henrique da Costa.

**Resources:** Mario Fritsch Neves.

**Supervision:** Thiago Prudente Bartholo.

**Writing – original draft:** Bruna Cuoco Provenzano, Thiago Prudente Bartholo, Claudia Henrique da Costa, Rogerio Rufino.

**Writing – review & editing:** Thiago Prudente Bartholo, Claudia Henrique da Costa, Rogerio Rufino.

## Supplementary Material


